# Strategic experimentation with asymmetric players

**DOI:** 10.1007/s00199-019-01193-9

**Published:** 2019-04-22

**Authors:** Kaustav Das, Nicolas Klein, Katharina Schmid

**Affiliations:** 1 University of Exeter Business School, Exeter, UK; 2 Université de Montréal and CIREQ, Montréal, Canada; 3 Gymnasium Bad Aibling Westendstraße 6A, 83043 Bad Aibling, Germany

**Keywords:** Two-armed bandit, Heterogeneous agents, Free riding, Learning, C73, D83, O31

## Abstract

We examine a two-player game with two-armed exponential bandits à la (Keller et al. in Econometrica 73:39–68, 2005), where players operate different technologies for exploring the risky option. We characterise the set of Markov perfect equilibria and show that there always exists an equilibrium in which the player with the inferior technology uses a cut-off strategy. All Markov perfect equilibria imply the same *amount* of experimentation but differ with respect to the expected speed of the resolution of uncertainty. If and only if the degree of asymmetry between the players is high enough, there exists a Markov perfect equilibrium in which both players use cut-off strategies. Whenever this equilibrium exists, it welfare dominates all other equilibria. This contrasts with the case of symmetric players, where there never exists a Markov perfect equilibrium in cut-off strategies.

## Introduction

In many instances, the information produced by one agent is interesting to other agents as well. Think, for example, of firms exploring neighbouring oil patches: if one firm strikes oil, chances are there will be oil in its neighbour’s patch as well. Such games of purely informational externalities have been analysed by the strategic bandit literature,^1^ which so far has only analysed the case of homogeneous agents. However, in many instances, one of the oil firms, for example, might be a big multinational firm that has access to a superior drilling technology. In this article, we aim to analyse the impact of asymmetries in players’ exploration technologies in a game of strategic experimentation with two-armed exponential bandits.

The seminal paper by Keller et al. (2005) analyses this problem with homogeneous players. In the current paper, we generalise the analysis by introducing asymmetric players, in the sense that their pay-off arrival rates from a good risky arm differ. This implies that, given the risky arm is good, the expected time needed to learn this differs between the players. As actions and outcomes are perfectly publicly observable, and players start out with a common prior, they will always have a common posterior belief. We characterise the set of Markov perfect equilibria with the players’ common posterior belief as the state variable for all ranges of asymmetry between the players. If the degree of asymmetry between the players is sufficiently high, there exists an *equilibrium in cut-off strategies*, i.e. where both players use a cut-off strategy. That is, either player uses the risky arm if and only if the likelihood he attributes to the option being good is greater than a certain threshold. This equilibrium is unique in the class of equilibria in cut-off strategies. Whenever only one of the players experiments and the other free rides in this equilibrium, it is always the player with the weaker technology who free rides. In the case of homogeneous players (Keller et al. 2005), by contrast, there never exists an equilibrium in cut-off strategies, and players swap the roles of pioneer and free rider at least once in any equilibrium. In our setting, aggregate pay-offs in the equilibrium in cut-off strategies are higher than in any other equilibrium. If the degree of asymmetry is low, at least one player uses a non-cut-off strategy in any equilibrium. In contrast to the homogeneous case (Keller et al. 2005), we furthermore show that more frequent switches of arms do not unambiguously improve the equilibrium welfare with asymmetric players.

### Related literature

This paper contributes to the literature on strategic experimentation with bandits, a problem studied quite widely in economics, amongst others, by Bolton and Harris (1999), Keller et al. (2005), Keller and Rady (2010), Klein and Rady (2011), Klein (2013) and Thomas (2017). In all of these papers, players are homogeneous. Except in Thomas (2017) and Klein and Rady (2011), players’ bandits are of the same type and * free riding* is a common feature in all the above models except for Thomas (2017). Many variants of this problem have been studied in the literature^2^. Rosenberg et al. (2013) and Murto and Välimäki (2011), for instance, assume that switches to the safe arm are irreversible and that experimentation outcomes are private information, while Bonatti and Hörner (2011) and Heidhues et al. (2015) investigate the case of private actions. In Dong (2018), actions and outcomes are public, but one of the players receives an initial private signal. Rosenberg et al. (2007) analyse the role of the observability of outcomes and the correlation between risky-arm types in a setting in which a switch to the safe arm is irreversible. Besanko and Wu (2013) use the Keller et al. (2005) framework to study how an R&D race is impacted by market structure. Das (2019) analyses an R&D race in a strategic bandit setting in which on the risky arm, players can learn both privately and publicly. Guo (2016) and Zambrano (2017) analyse the problem of a principal delegating the operation of a two-armed bandit to an agent; in Klein (2016), the bandit the agent operates has three arms. Banks et al. (1997) provide an experimental test of a single-agent two-armed bandit problem; Hoelzemann and Klein (2018) do so in a strategic setting. The paper closest to the present paper is Keller et al. (2005), who find that, with homogeneous players, there is never an equilibrium in cut-off strategies. By contrast, we show that, with heterogeneous players, an equilibrium in cut-off strategies may exist and that it is welfare maximising whenever it exists.

The rest of the paper is organised as follows. Section 2 sets out the model. Section 3 discusses the social planner’s solution. A detailed analysis of equilibria for different ranges of heterogeneity is undertaken in Sect. 4. Finally, Sect. 5 concludes. Payoff functions are shown in “Appendix A”, while some proofs are relegated to “Appendix B”.

## Two-armed bandit model with heterogeneous players

There are two players (1 and 2), each of whom faces a two-armed bandit in continuous time. One of the arms is safe, in that a player who uses it gets a flow pay-off of $s>0$. The risky arm can be either good or bad. Both players’ risky arms are of the same type. If the risky arm is good, then a player using it receives a lump sum, drawn from a time-invariant distribution with mean $h>s$, at the jumping times of a Poisson process. The Poisson process governing player 1’s arrivals has intensity $\lambda _1 = 1$, while player 2’s arrive according to a Poisson process with intensity $\lambda _2\in (\frac{s}{h},1)$. Thus, a good risky arm gives player 1 (2) an expected pay-off flow of $g_1=\lambda _1h = h$ ($g_2=\lambda _2h$), with $g_1>g_2>s$. The parameters and the game are common knowledge.

The uncertainty in this model arises from the fact that players do not initially know whether their risky arms are good or bad. Players start with a common prior belief $p_{0}\in (0,1)$ that their risky arms are good. Players have to decide in continuous time whether to choose the safe arm or the risky arm. At each instant, players can choose only one arm. We write $k_{i,t}=1$ ($k_{i,t}=0$) if player $i\in \{1,2\}$ uses his risky (safe) arm at instant $t\ge 0$. Players’ actions and outcomes are publicly observable, and based on these, they update their beliefs. Players discount the future according to the common discount rate $r>0$.

Let $p_t$ be the players’ common belief that their risky arms are good at time $t\ge 0$. Given player *i*’s ($i\in \{1,2\}$) actions $\{k_{i,t}\}_{t\ge 0}$, which are required to be progressively measurable with respect to the available information and to satisfy $k_i(t)\in \{0,1\}$ for all $t\ge 0$, player *i*’s expected pay-off is given by

$$\begin{aligned} \mathbb {E} \left[ \int _{0}^{\infty }r e^{-rt} [(1-k_{i,t})s + k_{i,t} p_t g_i ]\,\mathrm{d}t \right] , \end{aligned}$$

where the expectation is taken with respect to the processes $\{k_{i,t}\}_{t\ge 0}$ and $\{p_t\}_{t\ge 0}$. As can be seen from the objective function, there are no pay-off externalities between the players. Indeed, the presence of the other player impacts a given player’s pay-offs only via the information that he generates, i.e. via the belief.

As mentioned in Introduction, we will focus our analysis on Markov perfect equilibria with the players’ common posterior belief as the state variable. Formally, a Markov strategy of player *i* is any left-continuous function $k_i : [0,1] \rightarrow \{0,1\}, p\mapsto k_i(p)$ ($i = 1,2$) that is also piecewise continuous, i.e. continuous at all but a finite number of points.

As only a good risky arm can yield positive pay-offs in the form of lump sums, the arrival of a lump sum fully reveals the risky arm to be good. Hence, if either player receives a lump sum at a time $\tau \ge 0$, then $p_t = 1$ for all $t > \tau$. In the absence of a lump-sum arrival, the belief follows the following law of motion for a.a. *t*:

$$\begin{aligned} \mathrm{d}p_t = -(k_{1,t} +\lambda _2 k_{2,t})p_t (1-p_t) \, \mathrm{d}t. \end{aligned}$$

## Planner’s problem

Suppose there is a benevolent social planner, who controls the actions of both players and wants to maximise the sum of their pay-offs. Since the planner’s expected pay-off at any point in time only depends on the belief at that time and the belief follows a controlled Markov process, this is a Markov decision problem. Therefore, it is without loss of generality for the planner to restrict himself to Markov strategies $(k_1(p_t),k_2(p_t))$ with the posterior belief $p_t$ as the state variable. The Bellman equation for the planner’s problem is given by

1 $$\begin{aligned} v(p) = 2s + \max _{k_1,k_2 \in \{0,1\}} \big \{ k_1[ B_1(p,v) - c_1(p)] +k_2[B_2(p,v) - c_2(p) ] \big \}, \end{aligned}$$

where we write *v*(*p*) for the planner’s value function and, like Keller et al. (2005), define the myopic opportunity cost of having player *i* play risky, $c_i(p) = s - pg_i$, and the corresponding learning benefit

$$\begin{aligned} B_i(p,v) = p \frac{\lambda _i}{r}\{ (g_1+g_2) - v(p) - v^{^{\prime }}(p)(1-p)\}. \end{aligned}$$

Note that the planner’s Bellman equation is linear in both $$k_1$$ and $$ k_2$$, so that our restriction to action plans $$\{(k_{1,t},k_{2,t})\}_{t\ge 0}$$ with $$k_{i,t}\in \{0,1\}$$ for all (*i*, *t*) is without loss in the planner’s problem. To state the following proposition, which describes the planner’s solution, we define $$g = g_1+g_2$$, $$\lambda = 1+\lambda _2$$, $$\mu = \frac{r}{\lambda }$$, $$u_1(p): = (1-p)\left( \frac{1-p}{p}\right) ^r$$, $$u_0(p): =(1-p)\left( \frac{1-p}{p}\right) ^{\mu }$$.

### Proposition 1

The planner’s optimal policy $k^{*} (p)= (k_1^{*},k_2^{*})(p)$ is given by

$$\begin{aligned} (k_1^{*},k_2^{*}) (p)= \left\{ \begin{array}{lll} (1,1) &{}\quad \text{ if } p \in (p_2^{*},1) \\ (1,0) &{}\quad \text{ if } p \in (p_1^{*}, p_2^{*}] \\ (0,0) &{}\quad \text{ if } p \in (0, p_1^{*}] \end{array} \right. \end{aligned}$$

and the value function is

$$\begin{aligned} v(p) = \left\{ \begin{array}{lll} gp + \left[ \frac{\lambda }{\lambda _2}s-gp_2^*\right] \frac{u_0(p)}{u_0(p_2^*)} &{}\quad \text{ if } p \in (p_2^{*},1], \\ s + \left[ \frac{g + rg_1}{1+r} - \frac{s}{1+r}\right] p + \left[ s - \left( \frac{g + rg_1}{1+r} - \frac{s}{ 1+r}\right) p_1^{*}\right] \frac{u_1(p)}{u_1(p_1^*)} &{}\quad \text{ if } p \in (p_1^{*},p_2^{*}], \\ 2s &{}\quad \text{ if } p \in (0,p_1^{*}] , \end{array} \right. \end{aligned}$$

where $p_1^{*}$ is defined as

2 $$\begin{aligned} p_1^{*} = \frac{rs}{(1+r)g_1 + g_2 -2s}, \end{aligned}$$

and $p_2^{*}\in (p_1^*,\frac{s}{g_2})$ is implicitly defined by $v(p_2^{*}) = \frac{\lambda }{\lambda _2} s$.

### Proof

Proof is by a standard verification argument. Please see “Appendix B.1” for details. $\square$

By the above proposition, the belief at which player 1 switches to the safe arm in the planner’s solution is higher than it would be if both players’ Poisson arrival rates were equal to $\lambda _1=1$. This is because, as player 2’s arrival rate $\lambda _2$ decreases, the benefit from player 1’s experimentation decreases.

**Fig. 1:**
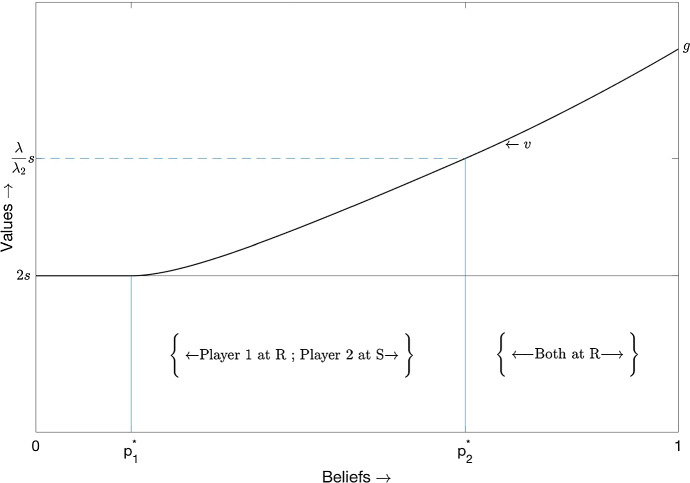
Planner’s solution

The planner’s solution is depicted in Fig. 1.^3^ The planner’s value function is a smooth convex curve which lies in the range [2*s*, *g*]. At the belief $p_2^{*}$($p_1^{*}$) , player 2 (1) switches to the safe arm.

## Non-cooperative game

We will first analyse a player’s best responses to a given Markov strategy of the other player.

### Best responses

Fix player *j*’s strategy $k_j$ ($j\in \{1,2\}\setminus \{i\}$). If the pay-off function from player *i*’s response satisfies the following Bellman equation, player *i* is playing a best response:^4^

3 $$\begin{aligned} v_i(p) = s+ k_j(p)\lambda _jb_i(p,v_i)+ \max _{k_i \in \{0,1\}} k_i[\lambda _i b_i(p,v_i) - (s - g_ip)] \end{aligned}$$

where

$$\begin{aligned} b_i(p,v_i) = p\frac{\{g_i-v_i-(1-p)v_i^{^{\prime }}\}}{r}. \end{aligned}$$

As before, $\lambda _i b_i(p,v_i)$ can be interpreted as the learning benefit accruing to player *i* due to his own experimentation, while $\lambda _j b_i(p,v_i)$ is the learning benefit accruing to player *i* from player *j*’s experimentation. The myopic opportunity cost of experimentation continues to be $c_i(p)=s-g_i p$.

For a given $k_j \in \{0,1\}$, from (3) we know that player *i*’s pay-off function satisfies the Bellman equation if and only if

$$\begin{aligned} k_i (p) \left\{ \begin{array}{lll} =1 &{}\quad \text {if } \lambda _ib_i(p,v_i) > s- g_ip, \\ \in \{0,1\} &{}\quad \text {if }\lambda _ib_i(p,v_i) = s-g_ip, \\ =0 &{}\quad \text {if} \lambda _ib_i(p,v_i) < s-g_ip. \end{array} \right. \end{aligned}$$

If $\lambda _ib_i(p,v_i) > s- g_ip$, then $k_i = 1$ is the unique best response. From 3, we can conclude that this requires $v_i> s +k_j \lambda _j b_i(p,v_i) > s+k_j\frac{\lambda _j}{\lambda _i} (s-g_ip)$. A similar argument applies for the situations when the best responses are $k_i \in \{0,1\}$ and $k_i = 0$, respectively. This allows us to infer that

$$\begin{aligned} k_i (p) \left\{ \begin{array}{lll} =1 &{}\quad \text {if } v_i > s + k_j \frac{\lambda _j}{\lambda _i}[s- g_ip], \\ \in \{0,1 \} &{}\quad \text {if }v_i = s + k_j \frac{\lambda _j}{\lambda _i}[s- g_ip] ,\\ =0 &{}\quad \text {if} v_i < s + k_j \frac{\lambda _j}{\lambda _i}[s- g_ip] . \end{array} \right. \end{aligned}$$

This implies that when $k_j = 1$, player *i* chooses the risky arm, safe arm or is indifferent between them depending on whether his value in the (*p*, *v*) plane lies above, below or on the line

4 $$\begin{aligned} D_i (p)= s+ \frac{\lambda _j}{\lambda _i}[s- g_ip] \end{aligned}$$

The single-agent threshold for player *i* is given by

5 $$\begin{aligned} \bar{p}_i = \frac{\mu _i s}{\mu _i s + (1+\mu _i) (g_i-s)} \end{aligned}$$

where $\mu _i = \frac{r}{\lambda _i}$. In “Appendix A.2”, we display the ODEs the players’ pay-off functions satisfy, as well as their solutions, for each possible action profile. We start off by showing that, as in the homogeneous case (Keller et al. 2005), no efficient equilibrium exists.

#### Proposition 2

In any MPE, both players play safe at all beliefs in $[0,\bar{p}_1]$. There is thus no efficient MPE.

#### Proof

Suppose to the contrary that $p_l$, the infimum of the set of beliefs at which at least one player plays risky satisfies $p_l < \bar{p}_1$. Clearly, $v_i(p_l)=s$ for both $i\in \{1,2\}$. We shall now distinguish two cases depending on whether or not there exists an $\bar{\epsilon }>0$ such that, in any $\epsilon$-right neighbourhood of $p_l$ with $\epsilon \in (0,\bar{\epsilon })$, only one player *i* plays risky. If there does not exist such an $\bar{\epsilon }>0$, *i* is not playing a best response, because $p_l<\bar{p}_i<\frac{s}{g_i}$ implies that the point $(p_l,s)$ is below the diagonal $D_i$. In the other case, player *i* faces the same trade-off as a single agent and does not play a best response either, because $p_l<\bar{p}_i$. $\square$

In the next subsection, we will characterise the condition under which an equilibrium in cut-off strategies exists.

### Equilibrium in cut-off strategies

As we have argued in the proof of Proposition 2, there is no experimentation below the belief $\bar{p}_1$ in any equilibrium. We will now argue that, in any equilibrium, only player 1 will experiment in some right neighbourhood of $\bar{p}_1$, implying that player 1 is the last player to experiment in any equilibrium.

By Proposition 2, we know that $v_1(\bar{p}_1)=v_2(\bar{p}_1)=s$, and thus, by continuity, both players’ value functions must be below their respective diagonals $D_i$ in some neighbourhood of $\bar{p}_1$. Thus, in any equilibrium, at most one player can play risky in some right neighbourhood of $\bar{p}_1$. Now, suppose that player 2 is the only player to experiment in some right neighbourhood of $\bar{p}_1$. Then, the relevant ODE (Equation 13 in “Appendix A.2”) gives us that $\lambda _2\bar{p}_1(1-\bar{p}_1)v_2'(\bar{p}_1+)=\bar{p}_1\lambda _2(g_2-s)-rc_2(\bar{p}_1)<0$, as $\bar{p}_1<\bar{p}_2$. Thus, player 2’s value function drops below *s* immediately to the right of $\bar{p}_1$, which contradicts his playing a best response. We can thus conclude that there exists some belief $\hat{p}_1>\bar{p}_1$ such that, on $(\bar{p}_1,\hat{p}_1)$, player 2 plays safe. As either player can always guarantee himself his single-agent pay-off by ignoring the information he gets for free from the other player, his pay-off in any equilibrium is bounded below by his single-agent pay-off. Thus, in any equilibrium, $v_1>s$ on $(\bar{p}_1,\hat{p}_1]$, and player 1 experiments, while player 2 free rides, in this range.

Thus, for beliefs right above $\bar{p}_1$, in any equilibrium, player 1’s pay-off is given by

6 $$\begin{aligned} \bar{v}_1(p) = g_1p + \bar{C}_1u_1(p), \end{aligned}$$

with $\bar{C}_1 = \frac{s-g_1\bar{p}_1}{u_1(\bar{p}_1)}$. Player 2’s equilibrium pay-off for these beliefs is given by

7 $$\begin{aligned} \bar{v}_2(p)= s + \frac{(g_2-s)p}{1+r} + \bar{C}_2 u_1(p) \end{aligned}$$

with $$\bar{C}_2 = -\frac{(g_2-s)\bar{p}_1}{(1+r)u_1(\bar{p}_1)}$$.

Since $\bar{C}_1 > 0$ and $\bar{C}_2 < 0$, $\bar{v}_1$ is strictly convex and $\bar{v}_2$ is strictly concave.^5^ The following lemma shows that the functions $\bar{v}_i$ intersect the corresponding diagonals $D_i$ at a unique belief.

#### Lemma 1

There exists a unique $p_1^{'} \in (\bar{p}_1, 1)$ such that $\bar{v}_1(p_1^{'}) = D_1(p_1^{'})$, and a unique $p_2^{'} \in \left( \bar{p}_2,\frac{s}{g_2}\right)$ such that $\bar{v}_2(p_2^{'}) = D_2(p_2^{'})$.

#### Proof

Please refer to “Appendix B.2”. $\square$

In the following proposition, we will show that there exists an equilibrium in cut-off strategies if and only if the degree of asymmetry between the players is high enough.

#### Proposition 3

There exists a $\lambda _2^{*} \in (\frac{s}{h}, 1)$ such that there exists an equilibrium in cut-off strategies if and only if $\lambda _2 \in (\frac{s}{h}, \lambda _2^{*}]$. In this equilibrium, player 1 plays risky on $(\bar{p}_1,1]$ and safe otherwise, while Player 2 plays risky on $(p_2^{'},1]$ and safe otherwise.

#### Proof

Please refer to “Appendix B.3”. $\square$

“Appendix B.4” shows that the belief $p_2^{'}$ where player 2 switches to the safe arm in the above equilibrium is strictly greater than $p_2^{*}$, the threshold in the planner’s solution. This shows that for $p \in (p_2^{*}, p_2^{'}]$, player 2 inefficiently free rides.

**Fig. 2:**
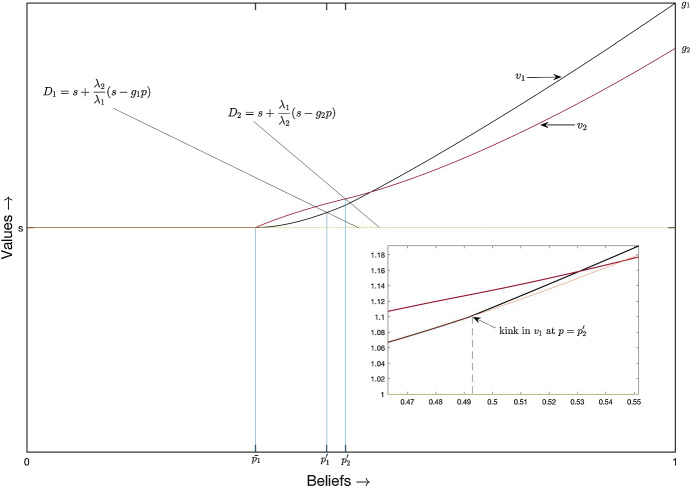
Equilibrium in cut-off strategies

The equilibrium in cut-off strategies is depicted in Fig. 2,^6^. In this equilibrium, both players’ pay-offs are equal to *s* for $p\le \bar{p}_1$. For $p > \bar{p}_1$, the black curve represents $v_1$ and the red curve represents $v_2$. For $p\in (\bar{p}_1, p_2^{'}]$, player *i*’s ($i=1,2$) pay-off is $\bar{v_i}(p)$. For $p>p_2^{'}$, player *i*’s pay-off is given by

$$\begin{aligned} v_i^r (p)= g_i p + C_i^r u_0(p) \end{aligned}$$

with $C_i^r = \frac{\bar{v_i}(p_2^{'}) - g_i p_2^{'}}{u_0(p_2^{'})}$.^7^ Player 1’s equilibrium pay-off function is (strictly) convex (on $(\bar{p}_1,1)$); it is smooth, except for a kink at $p_2^{'}$. (For the particular parameter values used in Fig. 2, we have $v_1^{'}(p_2^{'+}) = 1.477$ and $v_1^{'}(p_2^{'-}) = 1.21$). To depict this kink in the figure, we have magnified the area around $p = p_2^{'}$. In the magnified part, the orange curve represents $\bar{v_1}$ for $p>p_2^{'}$. Player 2’s pay-off function is strictly concave on $(\bar{p}_1,p_2^{'})$ and strictly convex on $(p_2^{'},1)$; it has an inflection point at $p_2^{'}$. It is smooth except for a kink at $\bar{p}_1$.

Experimentation decisions are strategic substitutes. Therefore in any equilibrium, at the lowest belief where some experimentation takes place, one *pioneer* is indifferent between choosing the safe and the risky arm, given that the other player is free riding. The *free rider* can determine a threshold belief $p_2^{'}$ where he is indifferent between choosing the safe arm and the risky arm, given that the pioneer is choosing the risky arm for all beliefs between the lowest cut-off and $p_2^{'}$. This implies that for beliefs just above $p_2^{'}$, the free rider finds it beneficial to experiment irrespectively of the action of the pioneer. When players are homogeneous, their free riding opportunities are the same. At $p_2^{'}$, the pioneer’s pay-off is less than that of the free rider as experimentation is costly. Thus, for beliefs just above $p_2^{'}$, the pioneer has an incentive to free ride, given that the free rider experiments. This explains [as shown in Keller et al. (2005)] why there does not exist an equilibrium where both players use cut-off strategies. However, the free riding opportunities are different for heterogeneous players. As explained above, in any equilibrium the pioneer is always the player with the higher productivity (player 1). The lower player 2’s productivity, the less player 1 has an incentive to free ride on player 2’s experimentation. If player 2’s productivity is very low, player 1 no longer has any incentive to free ride on 2’s experimentation for beliefs right above $p_2^{'}$. This intuitively explains the result of Proposition 3.

Geometrically, the diagonals $D_1$ and $D_2$ in Fig. 2 do not coincide when players are asymmetric. As the proof of Proposition 3 shows, the condition for existence of an equilibrium in cut-off strategies is precisely that player 2 will enter the region in which risky is dominant at a more optimistic belief than player 1.^8^ This is possible if and only if the region in which risky is dominant for player 2 is relatively small enough compared to that of player 1, i.e. if and only if $\lambda _2$ is small enough compared to $\lambda _1=1$.

In Sect. 4.5, we show that if the players’ learning speeds are different while the expected flow pay-off from the good risky arm is the same, there again exists an equilibrium in cut-off strategies if and only if the difference in the learning speeds is high enough. The same qualitative result obtains for identical learning speeds but different expected pay-offs from the good risky arm. Indeed, either form of asymmetry creates differences in the players’ free riding incentives. Diagrammatically, this can be seen by a gap between the best response diagonals.

### Equilibria in non-cut-off strategies

In the previous subsection, we have identified a necessary and sufficient condition for the existence of an equilibrium in cut-off strategies. In this subsection, we will analyse equilibria where at least one of the players uses a non-cut-off strategy. To begin with, we show that even for low degrees of asymmetry, there exists an equilibrium where player 2 uses a cut-off strategy.

#### Proposition 4

There exists an equilibrium in which only player 2 uses a cut-off strategy if and only if $\lambda _2>\lambda _2^*$. In this equilibrium, the cut-off for player 2’s strategy is $p_2^{'}$. Player 1 plays risky on $(\bar{p}_1, p_2^{'}] \cup (p_s^1, 1]$ and safe otherwise, where $p_s^1 > p_2^{'}$ is the belief at which player 1’s pay-off function and $D_1$ intersect.

#### Proof

Please refer to “Appendix B.5”. $\square$

**Fig. 3:**
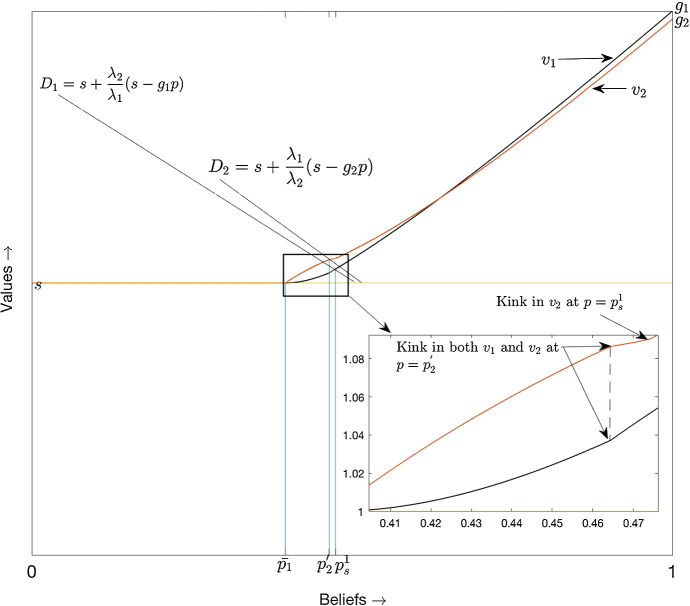
Only player 2 uses a cut-off strategy

The equilibrium where only player 2 uses a cut-off strategy is depicted in Fig. 3,^9^. The black and the orange curves depict the pay-offs to player 1 and 2, respectively. As the degree of asymmetry between the players is low, $p_1^{'} > p_2^{'}$, and hence, an equilibrium where both players use cut-off strategies does not exist. In Fig. 3, we magnify the part around $p = p_2^{'}$. We do not show $p_1^{'}$ in the figure, but for the parameter values used in Fig. 3, we have $p_s^1 = 0.4740 < 0.4754 = p_1^{'}$. At $p = p_2^{'}$, both $v_1$ and $v_2$ have a kink.^10^ To the immediate right of $p_2^{'}$, $v_1$ becomes concave and $v_2$ becomes convex. $v_2$ remains convex for all $p>p_2^{'}$, but has a kink^11^ at $p = p_s^1$. $v_1$ has an inflection point at $p = p_s^1$ and smoothly becomes convex at this belief.

Propositions 3 and 4 together imply that there always exists an equilibrium where player 2 uses a cut-off strategy with $p_2^{'}$ as the cut-off. Indeed, as argued in the previous subsection, in any equilibrium, $\bar{p}_1$ is the lowest belief where some experimentation takes place and only player 1 experiments at beliefs just above $\bar{p}_1$. By the same token, risky is Player 2’s best reply at all beliefs above $p_2^{'}$, given Player 1 plays risky on $(\bar{p}_1,p_2^{'}]$.

When the degree of asymmetry is low, there will exist a range of beliefs just above $p_2^{'}$ where player 1 free rides. Thus, player 1 uses a non-cut-off strategy. This explains the result of Proposition 4. In the limit $\lambda _2\downarrow \lambda _2^*$, the range above $p_2^{'}$ where player 1 free rides vanishes, and hence, the equilibrium described in Proposition 4 coincides with the equilibrium in cut-off strategies.

Equilibria where at least one player uses a non-cut-off strategy always exist, as the following proposition shows. The following proposition, together with Proposition 3, fully characterises the set of all Markov perfect equilibria. To state the proposition, we let $v_i$ be player *i*’s equilibrium pay-off. For both players $n\in \{1,2\}$, we define $p_S^n$ as the (unique) point of intersection of $v_n$ and $D_n$.^12^ Let $p_S^i=\min \{p_S^1,p_S^2\}$ and $p_S^j = \max \{p_S^1,p_S^2\}$.

#### Proposition 5

For any $\lambda _2\in (\frac{s}{h},1)$, there exists a continuum of Markov perfect equilibria in which at least one player uses a non-cut-off strategy. For each integer $l>1$ and each sequence of threshold beliefs $(\tilde{p}_i)_{i=1}^l$ such that $\bar{p}_1<\tilde{p}_1<\cdots <\tilde{p}_l=p_S^i$, there exists an equilibrium such that both players play safe at all beliefs $p\le \bar{p}_1$; player 1 plays risky and player 2 plays safe in $(\bar{p}_1,\tilde{p}_1]\cup \bigcup _{i\in 2\mathbb {N}\wedge i< l}(\tilde{p}_i,\tilde{p}_{i+1}]$ , while player 1 plays safe and player 2 plays risky in $\bigcup _{i\in 2\mathbb {N}\wedge i\le l}(\tilde{p}_{i-1},\tilde{p}_{i}]$; on $(p_S^i,p_S^j]$, player *i* plays risky and player *j* plays safe, while both players play risky on $(p_S^j,1]$. The same strategies with $l=1$ also describe an equilibrium in which only player 2 uses a cut-off strategy if and only if $p_2^{'}=p_S^2<p_S^1=\hat{p_S^1}$.

On $[0,\bar{p}_1]$, both players’ value function is *s*. For even $i<l$, on $(\tilde{p}_i,\tilde{p}_{i+1}]$, player 1’s (2’s) value function is given by (14), (16), while on $(\tilde{p}_{i-1},\tilde{p}_{i}]$, player 2’s (1’s) value function is given by (14), (16); on $(p_S^i,p_S^j]$, player *i*’s (*j*’s) pay-off is given by (14), (16). On $(p_S^j,1]$, both players’ pay-offs are given by (12). The constants of integration are determined by value matching.

#### Proof

That the proposed strategies are mutually best responses immediately follows from our discussion at the top of Sect. 4. That such equilibria always exist follows immediately from the continuity of players’ pay-off functions and the fact that $D_i(\bar{p}_1)>s$ for both $i\in \{1,2\}$. $\square$

When the degree of asymmetry is low, it is easy to observe that both players have incentives for free riding just below $p_2^{'}$; i.e. safe and risky are mutually best responses in this region. Although an increase in the degree of asymmetry reduces the free riding incentives for player 1, they never vanish completely. Therefore, there will always be a range just above $\bar{p}_1$ where safe and risky are mutually best responses. Hence, equilibrium allows players to take turns in experimenting at arbitrary beliefs in $( \bar{p}_1, p_2^{'})$. This explains the result of Proposition (5).

As $\bar{p}_1<\bar{p}_2$, the proposition implies that there exist equilibria in which player 2 experiments below his single-agent threshold $\bar{p}_2$. Indeed, by being the last player to experiment on $(\bar{p}_1,\tilde{p}_1]$, player 1 provides an *encouragement effect* to player 2, as the latter is willing to play risky on $(\tilde{p}_1,\tilde{p}_2]$ only because he knows that, should his experimentation not be successful, he will get to free ride on player 1’s experimentation once the belief will have dropped to $\tilde{p}_1$.

### Welfare rankings of equilibria

As in Keller et al. (2005), there are two potential sources of inefficiency in our model: players might not produce enough information, and/or they might produce the information too slowly. In order to analyse these different effects, we define the *experimentation intensity* at time $t \ge 0$ as $K_t = \lambda _1 k_{1,t}+ \lambda _2 k_{2,t}$, and the integral $\int _0^T K_t \, \mathrm{d}t$ as the *amount of experimentation* up to time *T*. Keller et al. (2005), by contrast, define the *experimentation intensity* at time $t \ge 0$ as $\hat{K}_t = k_{1,t}+ k_{2,t}$, and the *amount of experimentation* up to time *T* as $\int _0^T \hat{K}_t \, \mathrm{d}t$. Thus, we measure the *output* of players’ experimentation efforts, with our measure taking into account that it matters for the information-production process which player invests time in the risky arm. The corresponding concepts in Keller et al. (2005), by contrast, measure the *input*, i.e. the overall resources spent on producing information. In the case of homogeneous players with productivities $\lambda$, the input, as indicated by their measure, of course corresponds to $1/\lambda$ times the output, as indicated by our measure. The following result mirrors the finding in Keller et al. (2005) (see their Lemma 3.1 in conjunction with their Propositions 5.1 and 6.1) that the amount of experimentation is the same in any Markov perfect equilibrium. This implies that the welfare ranking of equilibria is solely determined by the delay in information production.

#### Lemma 2

Suppose there is no success on the risky arm. Then, the amount of experimentation is the same in any Markov perfect equilibrium.

#### Proof

As we have seen from our characterisation of equilibria, experimentation stops at $\bar{p}_1$ in any equilibrium. By Bayes’ rule, the law of motion of the belief conditional on no success is given by $\mathrm{d}p_t = -K_t p_t(1-p_t)\, \mathrm{d}t$. Thus, conditionally on no success, the amount of experimentation in any Markov perfect equilibrium is given by $\infty$ as upper bound

$$\begin{aligned} \int _0^\infty K_t \, \mathrm{d}t = \int _{p_0}^{\bar{p}_1} -\frac{\mathrm{d}p_t}{p_t(1-p_t)} = \left[ \ln \left( \frac{1-p}{p}\right) \right] _{p_0}^{\bar{p}_1}, \end{aligned}$$

which concludes the proof. $\square$

In the following proposition, we establish that in any equilibrium in which players swap the roles of pioneer and free rider at least once, player 1’s (2’s) pay-off will hit $D_1$ ($D_2$) at a more pessimistic (optimistic) belief than in the equilibrium in cut-off strategies.

#### Proposition 6

Consider any equilibrium described in Proposition 5. Suppose $p_S^1 >\bar{p}_1$ is the belief at which the equilibrium pay-off of player 1 meets the line $D_1$ and $p_S^2 > \bar{p}_1$ is the belief at which the equilibrium pay-off of player 2 meets the line $D_2$. Then, we have $p_S^1 < p_1^{'}$. For $l >1$ we have $p_S^2 > p_2^{'}$ and for $l=1$, $p_S^2 = p_2^{'}$.

#### Proof

Please refer to “Appendix B.6”. $\square$

In the equilibrium in cut-off strategies, player 2 free rides for all beliefs in $(\bar{p}_1, p_2^{'}]$. However, in any other equilibrium there exists some open subset of $(\bar{p}_1, p_2^{'})$ where he experiments and player 1 free rides. Thus, for all $p \in (\bar{p}_1, p_2^{'}]$, the equilibrium in cut-off strategies gives the highest pay-off to player 2, as he can free ride on the more productive player’s experimentation. This implies that, in the range $p \in (\bar{p}_1, p_2^{'}]$, player 2’s pay-off function in any non-cut-off equilibrium lies below his pay-off in the cut-off equilibrium and will therefore intersect the diagonal $D_2$ at a belief higher than $p_2^{'}$. This explains why we have $p_S^2 > p_2^{'}$. On the other hand, for all $p \in (\bar{p}_1, p_1^{'}]$, player 1 experiments in the equilibrium in cut-off strategies and receives his single-agent pay-off. In any other equilibrium, however, there exists some open subset of $(\bar{p}_1, p_1^{'})$ where his single-agent optimal action is not a best response, and his equilibrium pay-off is therefore higher. Thus, as player 1’s pay-off is lowest in the equilibrium in cut-off strategies, we have $p_S^1 < p_1^{'}$.

Suppose $\lambda _2 \in (\frac{s}{h},\lambda _2^{*}]$. This implies that the equilibrium in cut-off strategies exists. In the following proposition, we show that the equilibrium in cut-off strategies strictly welfare dominates all other equilibria.

#### Proposition 7

Suppose $\lambda _2\le \lambda _2^*$ and let $v_\mathrm{agg}^\mathrm{c}$ be the aggregate equilibrium pay-off in the equilibrium in cut-off strategies and $v_\mathrm{agg}^\mathrm{nc}$ be the aggregate equilibrium pay-off in an arbitrary equilibrium in non-cut-off strategies. Then, $v_\mathrm{agg}^\mathrm{c} \ge v_\mathrm{agg}^\mathrm{nc}$, with the inequality strict on $(\tilde{p}_1,1)$.

#### Proof

Please refer to “Appendix (B.7)”. $\square$

First, observe that in the equilibrium in cut-off strategies, both players experiment for beliefs greater than $p_2^{'}$. Since $p_S^2 > p_2^{'}$ (by Proposition 6), the range of beliefs where both players experiment is largest in the equilibrium in cut-off strategies. Next, in the equilibrium in cut-off strategies, whenever only one player experiments, it is the player with the higher pay-off arrival rate, player 1. In any other equilibrium, however, there is a range of beliefs where player 2 plays the role of the lonely pioneer. Since player 1 is more productive, in any equilibrium all experimentation ceases at $\bar{p}_1$, information is most efficiently generated in the equilibrium in cut-off strategies. This intuitively explains the result of Proposition 7. One can observe that, since, at any belief, the intensity of experimentation is highest in the equilibrium in cut-off strategies, information generation is fastest. Thus, this equilibrium involves least *delay*. As experimentation amounts are the same in all equilibria (Lemma 2), this implies that the cut-off equilibrium welfare dominates all other equilibria.^13^

**Fig. 4:**
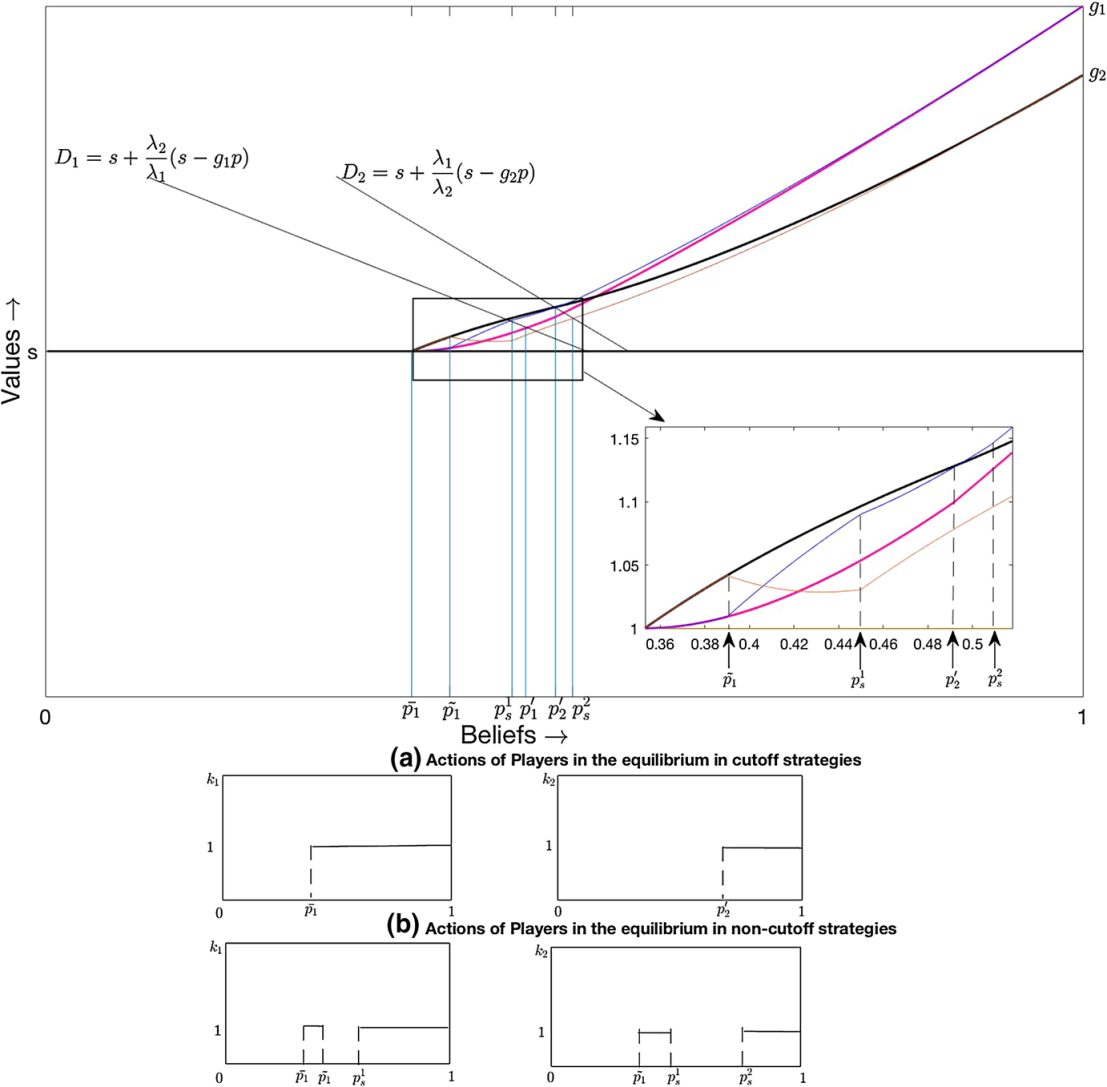
Comparison of cut-off equilibrium with equilibrium where players roles once

The comparison between the equilibrium in cut-off strategies and an equilibrium in which players swap roles once is depicted in Fig. 4.^14^ Figure 4a, b depicts the actions of players in the equilibrium in cut-off strategies and the equilibrium in non-cut-off strategies, respectively. These equilibria correspond to the ones depicted in Fig. 4.

The thick purple^15^curve ($v_1$) and the black curve ($v_2$) in Fig. 4 depict the pay-offs to player 1 and 2, respectively, in the equilibrium in cut-off strategies. In the equilibrium in non-cut-off strategies, pay-offs coincide for beliefs less than or equal to $\tilde{p_1}$. At $\tilde{p_1}$, players switch arms. The thin blue curve depicts the pay-off to player 1, and the thin yellow curve depicts the pay-off to player 2 in the equilibrium in non-cut-off strategies for $p>\tilde{p_1}$. As argued, the blue curve meets the line $D_1$ at a belief $p_S^1$, which is strictly less than $p_1^{'}$. In the region $(\tilde{p_1},p_S^1]$, player 2 experiments and player 1 free rides. At $p_s^1$, player 1 switches to the risky arm and player 2 switches to the safe arm. When the red curve meets the line $D_2$ at $p_s^2>p_2^{'}$, player 2 switches to the risky arm again. Notice that in the equilibrium in non-cut-off strategies, player 2’s pay-off is negatively sloped at the right neighbourhood of $p = \tilde{p_1}$. Indeed, in the current example, we have $\tilde{p_1} = 0.39 < 0.4054 = \bar{p}_2$, where $\bar{p}_2$ is the single person threshold for player 2. This means that, in the equilibrium in non-cut-off strategies, player 2 is forced to act as the lonely pioneer to the left of his single-agent cut-off, which makes his pay-off negatively sloped.^16^

When $\lambda _2 > \lambda _2^{*}$, the equilibrium in cut-off strategies does not exist. However, the argument in the proof of Proposition 7 allows us to show that, on $(\bar{p}_1,p_2^{'}]$, the equilibrium of Proposition 4, which is the only equilibrium in which player 1 is experimenting throughout this range, strictly welfare dominates all other equilibria. Indeed, with heterogeneous players, more frequent switches have the effect of replacing experimentation by the strong player with experimentation by the weak player in some open subset in $(\bar{p}_1,p_2^{'})$, thereby delaying information production in this range. Thus, even though more frequent switches can expand the range of beliefs where both experiment, there is always a welfare loss in the range $(\bar{p}_1, p_2^{'}]$. Hence, if players switch the role of pioneer and free rider more frequently, the equilibrium welfare is not unambiguously improved. This is in contrast to the case with homogeneous players (Keller et al. 2005), where the only effect of increasing the frequency of switches is to expand the range of beliefs where both players experiment, thus unambiguously speeding up information production and improving equilibrium welfare. Yet, we have not been able to establish that the equilibrium of Proposition 4 is *globally* welfare maximising.

### Learning rates versus pay-offs

In our baseline model, we have considered asymmetric Poisson arrival rates only. However, since the expected lump-sum pay-off from the good risky arm was the same for both players, the asymmetry in learning rates implied that the expected flow pay-off from a good risky arm was also different across the players. In this subsection, we will analyse a model where learning rates differ, but the expected flow pay-off from a good risky arm is the same for both players.

Define $\hat{g} = \lambda _1 h_1$ where $\lambda _1 = 1$ and $h_1 > 0$. For any $\lambda _2 \in (0,1)$, we choose a $h_2 > 0$ such that $\lambda _2 h_2 = \hat{g}$.

We will first analyse the social planner’s problem. Please refer to “Appendix (B.10)” for the explicit form of the Bellman equation for the planner’s value function *w*. The following proposition will show that the structure of the planner’s solution is the same as in Proposition 1.

#### Proposition 8

The planner’s optimal policy $k^{*} (p)= (k_1^{*},k_2^{*})(p)$ is given by

$$\begin{aligned} (k_1^{*},k_2^{*}) (p)= \left\{ \begin{array}{lll} (1,1) &{}\quad \text{ if } p \in (\bar{p}_2^{*},1) \\ (1,0) &{}\quad \text{ if } p \in (\bar{p}_1^{*}, \bar{p}_2^{*}] \\ (0,0) &{}\quad \text{ if } p \in (0, \bar{p}_1^{*}] \end{array} \right. \end{aligned}$$

and the value function is

$$\begin{aligned} w(p) = \left\{ \begin{array}{lll} 2\hat{g}p + \left[ \frac{\lambda }{\lambda _2}s-\hat{g}\bar{p}_2^{*}\frac{1-\lambda _2}{\lambda _2}-2\hat{g}\bar{p}_2^{*}\right] \frac{u_0(p)}{u_0(\bar{p}_2^{*})} &{}\quad \text{ if } p \in (\bar{p}_2^{*},1], \\ s + \left[ \frac{2\hat{g} + r\hat{g}}{1+r} - \frac{s}{1+r}\right] p + \left[ s - \left( \frac{2\hat{g} + r\hat{g}}{1+r} - \frac{s}{ 1+r}\right) \bar{p}_1^{*}\right] \frac{u_1(p)}{u_1(\bar{p}_1^{*})} &{}\quad \text{ if } p \in (\bar{p}_1^{*},\bar{p}_2^{*}], \\ 2s &{}\quad \text{ if } p \in (0,\bar{p}_1^{*}] , \end{array} \right. \end{aligned}$$

where $\bar{p}_1^{*}$ is defined as

8 $$\begin{aligned} \bar{p}_1^{*} = \frac{rs}{2(\hat{g}-s)+ r\hat{g}}, \end{aligned}$$

and $\bar{p}_2^{*}\in (\bar{p}_1^{*},\frac{s}{\hat{g}})$ is implicitly defined by $w(\bar{p}_2^{*}) = \frac{\lambda }{\lambda _2} s- \hat{g}\bar{p}_2^{*} \frac{1-\lambda _2}{\lambda _2}$.

#### Proof

Proof is by a standard verification argument. Please see the “Appendix B.8” for details. $\square$

We will now analyse the non-cooperative game. Please refer to “Appendix (B.10)” for the explicit form of the Bellman equation player *i*’s ($i = 1,2$) value function $w_i$ satisfies.

The single-agent thresholds are $\hat{p_i} = \frac{rs}{rs+(r+\lambda _i)(\hat{g}-s)}$. It can be verified that $\hat{p_1} < \hat{p_2}$. As in the baseline model, we can argue that in any equilibrium, $\hat{p_1}$ is the lowest belief where some experimentation takes place and player 1 is the last one to experiment. This implies that, in any equilibrium, for beliefs right above $\hat{p_1}$, pay-offs to player 1 and 2 are given by $\bar{w_1}(p)$ and $\bar{w_2}(p)$, respectively.^17^ It can be verified that $\bar{w_1}$ is strictly convex and $\bar{w_2}$ is strictly concave. By arguments similar to those in Lemma 1, we can infer that there exists a unique $\bar{p}_1^{'} \in (\hat{p_1},1)$ such that $\bar{w_1}(\bar{p}_1^{'}) = D_1(\bar{p}_1^{'})$ and a unique $\bar{p}_2^{'} \in (\hat{p_2}, \frac{s}{\hat{g}})$ such that $\bar{w_2}(\bar{p}_2^{'}) = D_2(\bar{p}_2^{'})$. In the following proposition, we establish that an equilibrium in cut-off strategies exists if and only if the degree of asymmetry is high enough.

#### Proposition 9

There exists a $\hat{\lambda }_2 \in (0,1)$ such that there exists an equilibrium in cut-off strategies if and only if $\lambda _2 \in (0,\hat{\lambda }_2]$. In this equilibrium, player 1 plays risky on $(\hat{p_1},1]$ and safe otherwise, while player 2 plays risky on $(\bar{p}_2^{'},1]$ and safe otherwise.

#### Proof

Please refer to “Appendix B.9” for details. $\square$

**Fig. 5:**
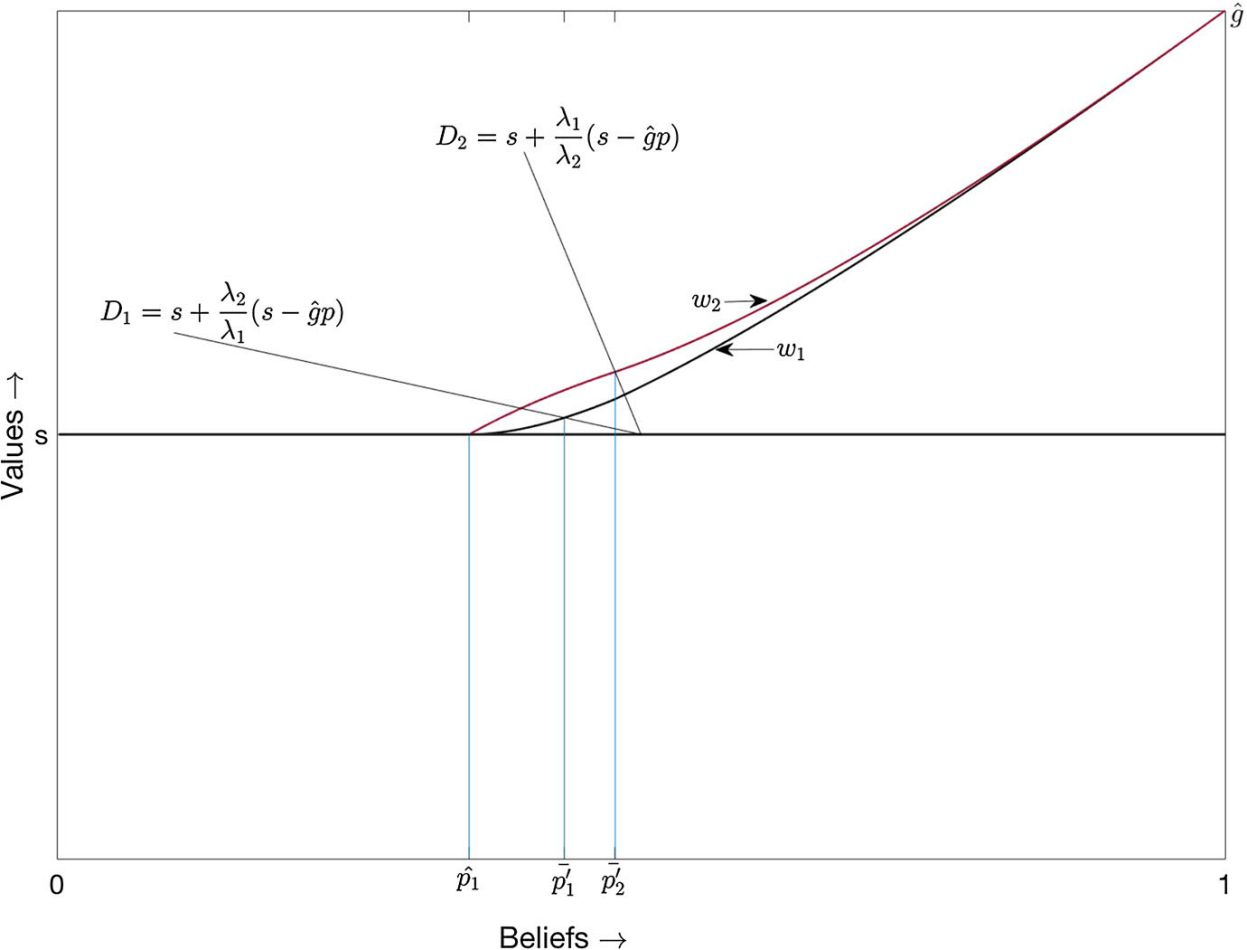
Cut-off equilibrium when only learning rates differ (color figure online)

Figure 5 depicts the equilibrium in cut-off strategies.^18^ The black (red) curve depicts the pay-offs to player 1 (2). Since the flow pay-off obtained by each player from a good risky arm is fixed at $\hat{g}$, the point of intersection between the best response line and the horizontal line $w = s$ is the same for both players. As agents become more asymmetric, the best response lines diverge more from each other. Due to this, there emerges a range of beliefs where only player 2 can free ride. Hence, if the degree of asymmetry between the players is high enough, there exists an equilibrium in cut-off strategies.

**Fig. 6:**
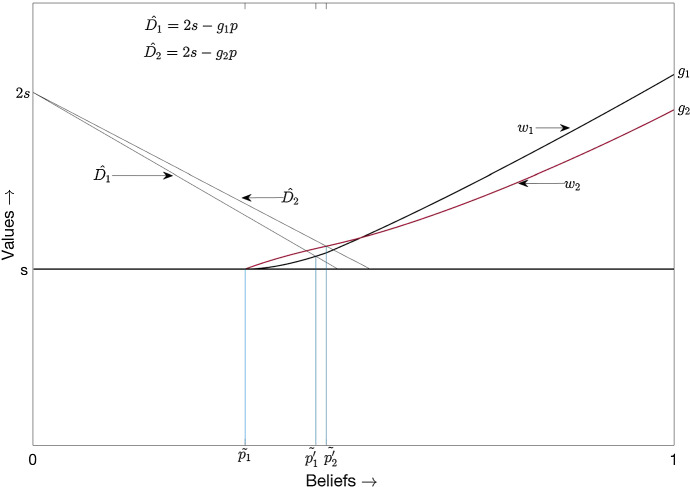
Cut-off equilibrium when learning rates are equal but risky flow pay-offs differ

Using similar arguments, we can establish that when the players’ learning rates are equal but their flow pay-offs from a good risky arm are different, an equilibrium in cut-off strategies exists if the asymmetry between the players is high enough. As an illustration, suppose $\lambda _1 = \lambda _2 =\hat{\lambda }$. The lump sum received by each player from a good risky arm at the jumping times of the Poisson process with intensity $\hat{\lambda }$ is drawn from a time-invariant distribution. The mean of this distribution $h_i$ ($i = 1,2$) is such that $h_1 > h_2$ and $h_2 \ge \frac{s}{\hat{\lambda }}$. This implies $g_1 > g_2 \ge s$. The best response diagonal of player *i* ($i=1,2$) is now given by $\hat{D}_i : v= 2s - g_i p$. Beliefs $\tilde{p_1^{'}}$ and $\tilde{p_2^{'}}$ can be defined analogously to $p_1^{'}$ and $p_2^{'}$ above. Figure 6,^19^ shows an equilibrium in cut-off strategies in this framework. The black (red) curve depicts the pay-offs to player 1 (2). This equilibrium exists only when the players are highly asymmetric, and the best response diagonals are far apart from each other.

In both cases, if it exists, the equilibrium in cut-off strategies is welfare maximising. The argument is similar to above: Player 2 free rides the most in the equilibrium in cut-off strategies, so that the range of beliefs at which both players play risky is largest. In addition, for any equilibrium that is not in cut-off strategies, there is an open set of beliefs in which the roles of experimenting pioneer and free rider are reversed as compared to the equilibrium in cut-off strategies (where only player 2 ever free rides). In the case $\lambda _1\not =\lambda _2$, both effects lead to greater delay in information production in the non-cut-off equilibrium. In the case $\lambda _1=\lambda _2=\hat{\lambda }$, the first effect leads to greater delay, while the second effect leads to a higher opportunity cost of information production ($s-g_1p < s-g_2p$), in the non-cut-off equilibrium.

## Conclusion

In this paper, we have characterised the set of Markov perfect equilibria in a two-armed bandit model with heterogeneous players. We have shown that there always exists an equilibrium in which the weaker player uses a cut-off strategy. If the heterogeneity is stark enough, there exists an equilibrium in cut-off strategies. If such an equilibrium exists, it is welfare optimal.

Thus, suppose there are two oil companies with vastly different drilling technologies, e.g. a big multinational firm and a small local enterprise. One could argue that the difference in technological capabilities between the two will be bigger in developing countries. On account of the big heterogeneity in capabilities, we should expect the equilibrium in cut-off strategies to exist. An empirically testable prediction of our model would thus be that there will be a higher frequency of instances in developing countries where the small local firm would free ride on the experimentation provided by the big multinational firm, and only enter the market after oil had been struck, even if the original level of uncertainty regarding the presence of oil was only moderate.

We have restricted players to using one arm only at any given instant *t*. By the linearity of the players’ Bellman equations, our equilibria would remain equilibria if we allowed players to select experimentation intensities $k_{i,t}\in [0,1]$. There might, however, be more equilibria in this case.

Our analysis has relied heavily on the characterisation of players’ best responses via the diagonals $D_i$ [see Eq. (4)], which was pioneered by Keller et al. (2005) for the homogeneous-player case. We expect that a similar approach could, *mutatis mutandis*, be used to study other kinds of asymmetries, e.g. pertaining to players’ safe-arm pay-offs $s_i$. We should expect a similar result to our Proposition 3 to hold in these cases, namely, that there existed an equilibrium in cut-off strategies if and only if the heterogeneity was stark enough.

## A Ordinary Differential Equations

### A.1 ODEs in the planner’s problem

Clearly, if $(k_1,k_2)=(0,0)$ is played at a belief *p*, the planner’s pay-off function satisfies $v(p)=2s$.

If the planner plays $k_1=k_2=1$ on an open set of beliefs, his pay-off function on this set satisfies

$$\begin{aligned} v(p) = 2s + B_1(p,v) - c_1(p) + B_2(p,v) -c_2(p), \end{aligned}$$

which is equivalent to the ODE

9 $$\begin{aligned} \lambda p (1-p)v^{'}(p) + (r+\lambda p)v(p) = (r+\lambda )pg. \end{aligned}$$

This is solved by

$$\begin{aligned} v(p) = gp + Cu_0(p) \end{aligned}$$

where *C* is a constant of integration.

By the same token, the ODE for $(k_1,k_2)=(1,0)$ is given by

10 $$\begin{aligned} p(1-p)v^{'}(p) + (r+p) v(p) = r(s+pg_1) + pg. \end{aligned}$$

This is solved by

$$\begin{aligned} v(p) = s+ \left[ \frac{g+rg_1}{1+r} - \frac{s}{1+r}\right] p + C u_1(p). \end{aligned}$$

### A.2 ODEs of players in the non-cooperative game

If $k_1=k_2=0$, both players’ pay-off functions satisfy $v_i(p)=s$.

If $k_1=k_2=1$ prevails on an open set of beliefs in the non-cooperative game, both players’ value function for beliefs in this set satisfies

11 $$\begin{aligned} \lambda p (1-p)v_i^{'}(p) + (r+\lambda p)v_i(p) = (r+\lambda )pg_i. \end{aligned}$$

This is solved by

12 $$\begin{aligned} v_i = g_i p + C u_0(p) \end{aligned}$$

where *C* is a constant of integration.

If $k_i=1$ and $k_j=0$, player *i*’s pay-off function satisfies

13 $$\begin{aligned} \lambda _i p (1-p)v_i^{'}(p) + (r+\lambda _i p)v_i(p) = (r+\lambda _i)pg_i. \end{aligned}$$

Solving this, we get

14 $$\begin{aligned} v_i(p) = g_i p + C u_i(p) \end{aligned}$$

where $u_i(p) = (1-p)[\frac{(1-p)}{p}]^{\mu _i}$ and $\mu _i=\frac{r}{\lambda _i}$. Player *j*’s pay-off function satisfies

15 $$\begin{aligned} \lambda _i p (1-p)v_j^{'}(p) + (r+\lambda _i p)v_j(p) = rs+\lambda _ipg_j. \end{aligned}$$

This is solved by

16 $$\begin{aligned} v_j = s + \frac{\lambda _i}{\lambda _i+r}(g_j-s) p + Cu_i(p). \end{aligned}$$

## B Proofs

### B.1 Proof of Proposition 1

The function *v* satisfies $v=2s$ on $[0,p_1^*]$, $v=2s+B_1-c_1$ on $(p_1^*,p_2^*]$ and $v=2s+B_1-c_1+B_2-c_2$ on $(p_2^*,1]$;^20^ thus, *v* is the pay-off function associated with the policy $k^*$.^21^ We shall first show that *v* is of class $C^1$, (strictly) increasing and (strictly) convex (on $(p_1^*,1)$).

One computes that, for $p\in (p_1^*,p_2^*)$, $B_1(p,v)-c_1(p)=\psi (p)$, where $\psi$ is defined as

$$\begin{aligned} \psi (p)= & {} -s+pg_1+\frac{1}{r}p\left[ g-s-\frac{g+rg_1}{1+r}+\frac{s}{1+r}\right. \\&\left. +\frac{r}{p}\left( s-p_1^*\left( \frac{g+rg_1}{1+r}-\frac{s}{r+1}\right) \right) \frac{u_1(p)}{u_1(p_1^*)}\right] . \end{aligned}$$

Direct computation shows that $u_1''>0$ and $s-p_1^*\left( \frac{g+rg_1}{1+r}-\frac{s}{1+r}\right) >0$, so that $\psi$, and hence $v\vert _{(p_1^*,p_2^*)}$, is strictly convex. One furthermore shows by direct computation that $\psi (p_1^*)=\psi '(p_1^*)=0$, implying that $v\vert _{(0,p_2^*)}$ is of class $C^1$.

We shall now show that $p_2^*$ is well-defined. Indeed, by definition, $x=p_2^*$ must satisfy

$$\begin{aligned} \left[ \frac{g + rg_1}{1+r} - \frac{s}{1+r}\right] x + \left[ s - \left( \frac{g + rg_1}{1+r} - \frac{ s}{1+r}\right) p_1^{*}\right] \frac{u_1(x)}{u_1(p_1^*)}= \frac{s}{\lambda _2} . \end{aligned}$$

The left-hand side of this equation is strictly increasing in *x* for $x>p_1^{*}$ and equal to $s<\frac{s}{\lambda _2}$ at $x=p_1^*$. Furthermore, at $x=\frac{s}{g_2}$, the left-hand side exceeds $\left[ \frac{g + rg_1}{1+r} - \frac{s}{1+r}\right] \frac{s}{g_2}>\frac{s}{\lambda _2}$. By continuity, the equation thus admits of a unique root $p_2^*\in (p_1^*,\frac{s}{g_2})$.

As $p_2^*<\frac{s}{g_2}$, $\frac{\lambda }{\lambda _2}s-p_2^*g>0$, and $v\vert _{[p_2^*,1]}$ is strictly convex as well. It remains to show that $v\vert _{[p_2^*,1]}$ is also strictly increasing. By convexity, it is sufficient to show smooth pasting at $p_2^*$. By the ODE for the region $(p_1^*,p_2^*)$ (Eq. 10 in “Appendix A.1”), we have $p_2^*(1-p_2^*)v'(p_2^*-)=\left[ rs+rp_2^*g_1+p_2^*g-(r+p_2^*)\frac{\lambda }{\lambda _2}s\right]$. By the ODE for the $(p_2^*,1)$-region (Equation 9 in “Appendix A.1”), we find $p_2^*(1-p_2^*)v'(p_2^*+)=\left[ (r+\lambda )p_2^*g-(r+\lambda p_2^*)\frac{\lambda }{\lambda _2}s\right] /\lambda$, and hence $v'(p_2^*+)=v'(p_2^*-)$.

It remains to show that *v* solves the Bellman equation, i.e. that $B_i\le c_i$ for both $i\in \{1,2\}$ on $[0,p_1^*]$; $B_1\ge c_1$ and $B_2\le c_2$ on $(p_1^*,p_2^*]$; and $B_i\ge c_i$ for both $i\in \{1,2\}$ on $(p_2^*,1]$. First, let $p\in [0,p_1^*]$. In this case, $v=2s$, and $B_i\le c_i$ if and only if $p\le \frac{rs}{rg_i+\lambda _i(g-2s)}$, which is verified for all $p\le p_1^*$. Now, let $p\in (p_1^*,p_2^*]$. As *v* is strictly increasing in this range, $v=2s+B_1-c_1>2s$, and thus $B_1>c_1$. Moreover, $v=2s+B_1-c_1$ implies that $B_2=\lambda _2B_1=\lambda _2\left( v-s-pg_1\right) \le s-pg_2=c_2$ if and only if $v\le \frac{\lambda }{\lambda _2}s$, which is verified as $p\le p_2^*$. Finally, let $p\in (p_2^*,1)$. In this range, we have that $g-v-(1-p)v'=\frac{r}{\lambda p}\left( \frac{\lambda }{\lambda _2}s-p_2^*g\right) \frac{u_0(p)}{u_0(p_2^*)}$, so that $B_i=\frac{\lambda _i}{\lambda }v-pg_i$, which exceeds $c_i=s-pg_i$ if and only if $v\ge \frac{\lambda }{\lambda _i}s$. By monotonicity of *v*, $v\ge \frac{\lambda }{\lambda _2}s>\lambda s$ in this range, which completes the proof.

### B.2 Proof of Lemma 1

The function $\bar{v}_1$ is strictly increasing, while $D_1$ is strictly decreasing. Furthermore, $\bar{v}_1(\bar{p}_1)<D_1(\bar{p}_1)$ and $\bar{v}_1(1)>D_1(1)$. Since both $\bar{v}_1$ and $D_1$ are moreover continuous, there exists a unique $p_1^{'} \in (\bar{p}_1, 1)$ such that $\bar{v}_1(p_1^{'}) = D_1(p_1^{'})$.

As $\bar{C}_2<0$, we have

$$\begin{aligned} \bar{v}_2(\bar{p}_2) < s+ \frac{[g_2-s]\bar{p}_2}{1+r} = s+\frac{[g_2-s]}{1+r}\frac{\mu _2 s}{(\mu _2+1)g_2 - s}\equiv \bar{\varPsi }. \end{aligned}$$

On the other hand, $D_2(\bar{p}_2) = s+ \frac{s[g_2-s]}{\lambda _2[(\mu _2+1)g_2-s]}$. This implies

$$\begin{aligned} D_2(\bar{p}_2) - \bar{\varPsi } = \frac{s[g_2-s]}{\left[ (\mu _2+1)g_2-s\right] \lambda _2( 1+r)} > 0. \end{aligned}$$

Hence, $D_2(\bar{p}_2) > \bar{v}_2(\bar{p}_2)$. Furthermore, the function $\bar{v}_2$ is strictly increasing, and $D_2$ is strictly decreasing, on $\left( \bar{p}_2,\frac{s}{g_2}\right)$, while $\bar{v}_2 \left( \frac{s}{g_2}\right) > D_2\left( \frac{s}{g_2}\right) =s$. Since both $\bar{v}_2$ and $D_2$ are moreover continuous, there exists a unique $p_2^{'} \in \left( \bar{p}_2,\frac{s}{g_2}\right)$ such that $\bar{v}_2(p_2^{'}) = D_2(p_2^{'})$.

### B.3 Proof of Proposition 3

#### Proof

By our previous arguments, in any equilibrium in cut-off strategies, player 1 will play risky on $(\bar{p}_1,1]$ and safe otherwise. In response, by the definition of $p_2^{'}$, player 2 must play risky on $(p_2^{'},1]$ and safe otherwise, if there is an equilibrium in cut-off strategies. Indeed, below $p_2^{'}$, player 2 is playing a best response to player 1’s action choice by the definition of $p_2^{'}$. Since $D_2$ is decreasing, it is sufficient to show that player 2’s pay-off function is increasing on $[p_2^{'},1]$ in order to show that he is also playing a best response at beliefs above $p_2^{'}$. Firstly, we note that the closed-form expression for player 2’s pay-off function (see Eq. 12 in “Appendix A.2”) implies that player 2’s pay-off $v_2$ is strictly convex on $(p_2^{'},1)$, as $v_2(p_2^{'})=D_2(p_2^{'})>g_2p_2^{'}$, where the inequality follows from $p_2^{'}<\frac{s}{g_2}$ (see Lemma 1). Furthermore, the relevant ODEs (Eqs. 15 and 11 in “Appendix A.2”) show that $v_2(p_2^{'})=D_2(p_2^{'})$ implies smooth pasting at $p_2^{'}$. As moreover $\bar{v}_2'>0$ (as $\bar{C}_2<0$ and $u_1'<0$), we can conclude that player 2’s value function is strictly increasing on $(p_2^{'},1)$ as well, and hence that player 2 is playing a best response at beliefs above $p_2^{'}$.

Thus, the candidate strategy profile is indeed an equilibrium if and only if player 1’s strategy is a best response to player 2’s. This requires player 2 to choose the safe arm for all beliefs at which player 1’s pay-off is below $D_1$. Thus, it remains to determine under what conditions $p_2^{'} \ge p_1^{'}$.

We will first argue that $p_1^{'}$ ($p_2^{'}$) is increasing (decreasing) in $\lambda _2$. Recall that $p_1^{'}$ is the point of intersection of the function $\bar{v_1}$ and the line $D_1$. As $\lambda _2$ decreases, the line $D_1$ rotates anticlockwise around the point $(\frac{s}{g_1}, s)$. Since $\bar{v_1}$ is independent of $\lambda _2$, $p_1^{'}$ decreases as $\lambda _2$ decreases. On the other hand, as $\lambda _2$ decreases, the line $D_2$ shifts to the right and becomes steeper. By direct computation, one shows that $\bar{v_2}$ becomes flatter as $\lambda _2$ decreases. This implies that $p_2^{'}$ increases.

Consider the case $\lambda _2 \downarrow \frac{s}{h}$. Then, $D_2\rightarrow s+\frac{(s-sp)}{\lambda _2}$. Thus, the belief $\hat{p}$ such that $D_2(\hat{p})=s$ will tend to 1. Moreover, $\bar{v}_2\rightarrow s$. Hence, $p_2^{'} \rightarrow 1$. However, $D_1$ still intersects the line *s* at $p = \frac{s}{g_1}$, implying that $p_1'\le \frac{s}{g_1} < p_2^{'}$.

Next, we consider the case $\lambda _2\uparrow 1$ and argue that there exists a left neighbourhood of 1 such that, for all $\lambda _2$ in this neighbourhood, $p_2^{'} < p_1^{'}$. Now, suppose that $\bar{v}_1(p^\dagger )=\bar{v}_2(p^\dagger )$ while $\bar{v}'_1(p^\dagger )\ge \bar{v}'_2(p^\dagger )$ for some $p^\dagger$ in the interior of the (1, 0)-region. The relevant ODEs then imply that $p^\dagger \ge \check{p}=\frac{rs}{rg_1+(g_1-g_2)}$. It thus follows that $\bar{v}_2>\bar{v}_1$ for all beliefs in $(\bar{p}_1,\check{p}]$. Note that $\check{p}<\frac{s}{g_1}$ and $\check{p} \uparrow \frac{s}{g_1}$ as $\lambda _2 \uparrow 1$. Furthermore, recall that $p_1'$ is implicitly defined by

$$\begin{aligned} (1+\lambda _2)(g_1p_1'-s)+\bar{C}_1u_1(p_1')=0, \end{aligned}$$

where we note that $\bar{C}_1$ and $u_1$ are both independent of $\lambda _2$. This implies that (1) $p_1'<\frac{s}{g_1}$ for all $\lambda _2\in [\frac{s}{h},1]$ (as $\bar{C}_1>0$ and $u_1 > 0$ for $p<1$), and (2) that $p_1'$ is a continuous function of $\lambda _2$ (by the Implicit Function Theorem). Therefore $\hat{p}=\max _{\lambda _2\in [\frac{s}{h},1]}p_1'<\frac{s}{g_1}$. Thus, we can choose $\underline{\lambda }_2\in (\frac{s}{h},1)$ such that, for all $\lambda _2\in [\underline{\lambda }_2,1]$, $\check{p}>\hat{p}$, and therefore $\bar{v}_2>\bar{v}_1$ on $(\bar{p}_1,p_1']$. It thus follows that, for $\lambda _2\in [\underline{\lambda }_2,1]$, $\tilde{p}_2<p_1'$, where $\tilde{p}_2$ is the belief where the function $\bar{v_2}$ intersects the line $D_1$. As $p_2'\downarrow \tilde{p}_2$ for $\lambda _2\uparrow 1$, we can conclude that there exists some $\underline{\hat{\lambda }}_2\in (\frac{s}{h},1)$ such that, for all $\lambda _2\in (\underline{\hat{\lambda }}_2,1)$, $p_2'<p_1'$. Thus, by monotonicity of $p_1^{'}$ and $p_2^{'}$ in $\lambda _2$, there exists a unique $\lambda _2^{*} \in (\frac{s}{h}, 1)$ such that $p_2^{'} \ge p_1^{'}$ if and only if $\lambda _2 \in (\frac{s}{h}, \lambda _2^{*}]$. $\square$

### B.4 To show that $$p_2^{*} < p_2^{'} $$

Recall from the proof of Proposition 8 that $p_2^*$ was implicitly defined as the unique root of the (for $p>p_1^{*}$) strictly increasing function $\zeta$, where

$$\begin{aligned} \zeta (p)=\left[ g_1+\frac{g_2-s}{1+r}\right] p+\left[ s-\left( g_1+\frac{g_2-s}{1+r}\right) p_1^*\right] \frac{u_1(p)}{u_1(p_1^*)}-\frac{s}{\lambda _2}. \end{aligned}$$

By the same token, $p_2^{'}$ is implicitly defined by $\bar{v}_2(p_2^{'})=D_2(p_2^{'})$, which is equivalent to

$$\begin{aligned} \frac{g_2-s}{1+r}p_2^{'}+p_2^{'}g_1-\frac{s}{\lambda _2}=\frac{g_2-s}{1+r}\bar{p}_1\frac{u_1(p_2^{'})}{u_1(\bar{p}_1)}. \end{aligned}$$

As $p_2^{'}>\bar{p}_2>p_1^*$, it remains to show that

$$\begin{aligned} \zeta (p_2^{'})=\frac{g_2-s}{1+r}\bar{p}_1\frac{u_1(p_2^{'})}{u_1(\bar{p}_1)}+ \left[ s-\left( g_1+\frac{g_2-s}{1+r}\right) p_1^*\right] \frac{u_1(p_2^{'})}{u_1(p_1^*)}> 0. \end{aligned}$$

For this, it is sufficient that

$$\begin{aligned} s-\left( g_1+\frac{g_2-s}{1+r}\right) p_1^{*}>0, \end{aligned}$$

which follows by direct computation.

### B.5 Proof of Proposition 4

If $\lambda _2\le \lambda _2^*$, $p_1^{'}\le p_2^{'}$, by the proof of Proposition 3. Suppose to the contrary that the equilibrium in which only player 2 uses a cut-off exists. By Proposition 6, $p_S^1<p_1^{'}\le p_2^{'}=p_S^2$, a contradiction to the characterisation of this equilibrium in Proposition 5.

Now, suppose $\lambda _2>\lambda _2^*$. By the proof of Proposition 3, $p_1^{'}> p_2^{'}$. It thus remains to show that $\hat{p_S^1}>p_2^{'}$. Yet, player 1’s pay-off from the conjectured equilibrium strategies at $p_2^{'}$ is given by $\bar{v}_1(p_2^{'})<D_1(p_2^{'})$, the inequality being immediately implied by $p_1^{'}> p_2^{'}$, we have $\hat{p_S^1}>p_2^{'}$, and, by Proposition 5, the equilibrium exists.

### B.6 Proof of Proposition 6

First, consider $l> 1$. It is sufficient to show that $\bar{v}_1<v_1$ and $\bar{v}_2>v_2$ on $(\tilde{p}_1,p_S^j]$, where $v_n$ is player *n*’s equilibrium pay-off function.

Note that $\bar{v}_n(\tilde{p}_1)=v_n(\tilde{p}_1)$ for both $n\in \{1,2\}$ and suppose that $\bar{v}_2(\tilde{p}_{i-1})\ge v_2(\tilde{p}_{i-1})$ and $\bar{v}_1(\tilde{p}_{i-1})\le v_1(\tilde{p}_{i-1})$ for some $i\in \{2,\cdots ,k\}$. Suppose that $i-1\ge 1$ is odd, and let $v_1^{rr}$ be player 1’s pay-off from deviating to playing risky on $(\tilde{p}_{i-1},\tilde{p}_i]$. By construction, $v_1^{rr}(\tilde{p}_{i-1})=v_1(\tilde{p}_{i-1})\ge \bar{v}_1(\tilde{p}_{i-1})$. Suppose to the contrary that there exists a belief $p\in (\tilde{p}_{i-1},\tilde{p}_i]$ such that $\bar{v}_1(p)=v_1^{rr}(p)$. The relevant ODEs [(13) and (11)] imply that $v_1^{rr^{'}}(p-)>\bar{v}_1'(p-)$. As $v_1^{rr}(\tilde{p}_{i-1})=v_1(\tilde{p}_{i-1}) \ge \bar{v}_1(\tilde{p}_{i-1})$, this implies that there exists a $\hat{p}\in [\tilde{p}_{i-1},\tilde{p}_i)$ such that $v_1^{rr}(\hat{p})=\bar{v}_1(\hat{p})$ and $v_1^{rr^{'}}(\hat{p}+)<\bar{v}_1'(\hat{p}+)$, a contradiction to (13) and (11). By the same token, suppose that there exists a belief $p\in (\tilde{p}_{i-1},\tilde{p}_i]$ such that $v_2(p)=\bar{v}_2(p)$. As $s>pg_2$, the relevant ODEs ((13) and (15)) imply that $\bar{v}_2^{'}(p-)>v_2'(p-)$. As $\bar{v}_2(\tilde{p}_{i-1})\ge v_2(\tilde{p}_{i-1})$, this implies that there exists a $\hat{p}\in [\tilde{p}_{i-1},\tilde{p}_i)$ such that $v_2(\hat{p})=\bar{v}_2(\hat{p})$ and $v_2^{'}(\hat{p}+)>\bar{v}_2'(\hat{p}+)$, a contradiction to (13) and (15).

Now, let $i-1\ge 2$ be even. Note that our previous step implies that $\bar{v}_2(\tilde{p}_{i-1})>v_2(\tilde{p}_{i-1})$ and $\bar{v}_1(\tilde{p}_{i-1})<v_1(\tilde{p}_{i-1})$. Suppose that there exists a $p\in (\tilde{p}_{i-1},\tilde{p}_i]$ such that $v_n(p)=\bar{v}_n(p)$ for an $n\in \{1,2\}$. As $(k_1,k_2)=(1,0)$ on $(\tilde{p}_{i-1},\tilde{p}_i]$, this immediately implies that $v_n(\tilde{p}_{i-1})=\bar{v}_n(\tilde{p}_{i-1})$, a contradiction.

On $(\tilde{p}_k,p_S^j]$, a similar argument to the case of even (odd) $i-1$ applies if $j=2$ ($j=1$), so that we can conclude that $\bar{v}_1<v_1$ and $\bar{v}_2>v_2$ on $(\bar{p}_1,p_S^j]$, and hence $p_S^1<p_1^{'}$ and $p_S^2>p_2'$.

For $l = 1$, from the equilibrium characterisation we know that $p_S^2 = p_2^{'}$ and the above argument to show $p_S^1 < p_1^{'}$ still applies.

### B.7 Proof of Proposition 7

If player *i* ($i=1,2$) experiments and player *j* ($j=1,2$, $j\ne i$) free rides then the players’ aggregate equilibrium pay-off is given by $v_\mathrm{agg} = v_i +v_j$, with $v_i$ satisfying the ODE (13) and $v_j$ satisfying the ODE (15). If both players experiment then $v_\mathrm{agg} = v_1+v_2$ and $v_n$ ($n=1,2$) satisfy the ODE (11).

From Proposition 5, we know that $v_\mathrm{agg}^\mathrm{c}(\tilde{p}_1) = v_\mathrm{agg}^\mathrm{nc}(\tilde{p}_1)$. Suppose $v_\mathrm{agg}^\mathrm{c}(\tilde{p}_{i-1}) \ge v_\mathrm{agg}^\mathrm{nc}(\tilde{p}_{i-1})$ for some $i \in \{2,3,\ldots ,k\}$. Suppose first that $i-1 \ge 1$ is odd. If there exists a $p \in (\tilde{p}_{i-1}, \tilde{p}_i]$ such that $v_\mathrm{agg}^\mathrm{c}(p) = v_\mathrm{agg}^\mathrm{nc}(p)$, then by the ODEs (13) and (15), we can conclude that $v_\mathrm{agg}^\mathrm{c'}(p-) > v_\mathrm{agg}^\mathrm{nc'}(p-)$. This implies there exists a $\hat{p} \in [\tilde{p}_{i-1}, p)$ such that $v_\mathrm{agg}^\mathrm{c}(\hat{p}) = v_\mathrm{agg}^\mathrm{nc}(\hat{p})$ and $v_\mathrm{agg}^\mathrm{c'}(\hat{p}+) < v_\mathrm{agg}^\mathrm{nc'}(\hat{p}+)$, a contradiction to ODEs (13) and (15).

Suppose $i-1 \ge 2$ is even. Then from the previous step we can infer that $v_\mathrm{agg}^\mathrm{c}(\tilde{p}_{i-1}) > v_\mathrm{agg}^\mathrm{nc}(\tilde{p}_{i-1})$. In both kinds of equilibria, if $i-1$ is even, $(k_1,k_2) = (1,0)$ on $(\tilde{p}_{i-1},\tilde{p}_i]$. This implies that we have $v_\mathrm{agg}^\mathrm{c}(p) > v_\mathrm{agg}^\mathrm{nc}(p)$ for all $p \in (\tilde{p}_{i-1}, \tilde{p}_{i}]$. Thus, for all $p \in (\tilde{p}_1, \tilde{p}_k]$, $v_\mathrm{agg}^\mathrm{c} (p)> v_\mathrm{agg}^\mathrm{nc}(p)$.

As $\lambda _2\le \lambda _2^*$, we have $\tilde{p}_k = p_S^1$. An argument similar to that for even $i-1$ shows that $v_\mathrm{agg}^\mathrm{c} > v_\mathrm{agg}^\mathrm{nc}$ on $p \in (p_S^1, p_2^{'}]$. Now, suppose that there exists a $\hat{p}\in (p_2^{'},p_S^2]$ such that $v_\mathrm{agg}^\mathrm{c} (\hat{p})= v_\mathrm{agg}^\mathrm{nc}(\hat{p})$. By the ODEs (13) and (11), this implies $v_\mathrm{agg}^\mathrm{c'} (\hat{p}-)> v_\mathrm{agg}^\mathrm{nc'}(\hat{p}-)$. This leads to a contradiction by the same argument as above. As $(k_1,k_2)=(1,1)$ prevails in both equilibria on $(p_S^2,1)$, the claim follows.

### B.8 Proof of Proposition 8

The function *w* satisfies $w=2s$ on $[0,\bar{p}_1^*]$, $v=2s+\bar{B}_1-\hat{c}$ on $(\bar{p}_1^*,\bar{p}_2^*]$ and $w=2s+\bar{B}_1-\bar{c}+\bar{B}_2-\bar{c}$ on $(\bar{p}_2^*,1]$; thus, *w* is the pay-off function associated with the policy $k^*$.^22^ We shall first show that *w* is of class $C^1$, (strictly) increasing and (strictly) convex (on $(\bar{p}_1^*,1)$).

As in Proposition 1, we can compute for $p\in (\bar{p}_1^*,\bar{p}_2^*)$, $\bar{B}_1(p,v)-\bar{c}(p)=\phi (p)$, where $\phi$ is defined as

$$\begin{aligned} \phi (p)= & {} -s+p\hat{g}+\frac{1}{r}p\left[ 2\hat{g}-s-\frac{2\hat{g}+r\hat{g}}{1+r}+\frac{s}{1+r}\right. \\&\left. +\frac{r}{p}\left( s-\bar{p}_1^*\left( \frac{2\hat{g}+r\hat{g}}{1+r}-\frac{s}{r+1}\right) \right) \frac{u_1(p)}{u_1(\bar{p}_1^*)}\right] . \end{aligned}$$

Direct computation shows that $u_1''>0$ and $s-\bar{p}_1^*\left( \frac{2\hat{g}+r\hat{g}}{1+r}-\frac{s}{1+r}\right) >0$, so that $\phi$, and hence $w\vert _{(\bar{p}_1^*,\bar{p}_2^*)}$ is strictly convex. One furthermore shows by direct computation that $\phi (\bar{p}_1^*)=\phi '(\bar{p}_1^*)=0$, implying that $w\vert _{(0,\bar{p}_2^*)}$ is of class $C^1$.

We shall now show that $\bar{p}_2^*$ is well-defined. Indeed, by definition, $x=\bar{p}_2^*$ must satisfy

$$\begin{aligned}&s+ \left[ \frac{2\hat{g} + r\hat{g}}{1+r} - \frac{s}{1+r}\right] x + \left[ s - \left( \frac{2\hat{g} + r\hat{g}}{1+r} - \frac{ s}{1+r}\right) \bar{p}_1^{*}\right] \frac{u_1(x)}{u_1(\bar{p}_1^*)} \\&\quad + \frac{\hat{g}[1-\lambda _2]}{\lambda _2}x = \frac{\lambda }{\lambda _2}s . \end{aligned}$$

The left-hand side of this equation is strictly increasing in *x* for $x>\bar{p}_1^{*}$ and equal to $s + \frac{s}{\lambda 2}\frac{[\hat{g}(2\lambda _2+r)-2\lambda _2s]}{[\hat{g}(2+r) - 2s]}<\frac{\lambda }{\lambda _2}s$ at $x=\bar{p}_1^*$. Furthermore, at $x=\frac{s}{\hat{g}}$, the left-hand side exceeds $s + \left[ \frac{2\hat{g} + r\hat{g}}{1+r} - \frac{s}{1+r}\right] \frac{s}{\hat{g}}>\frac{\lambda }{\lambda _2}s$. By continuity, the equation thus admits of a unique root $\bar{p}_2^*\in (\bar{p}_1^*,\frac{s}{\hat{g}})$.

As $\bar{p}_2^*<\frac{s}{\hat{g}}$, $\frac{\lambda }{\lambda _2}s- \frac{\hat{g}[1-\lambda _2]}{\lambda _2}\bar{p}_2^* - 2\hat{g}\bar{p}_2^*>0$, and $w\vert _{[\bar{p}_2^*,1]}$ is strictly convex as well. It remains to show that $w\vert _{[\bar{p}_2^*,1]}$ is also strictly increasing. By convexity, it is sufficient to show smooth pasting at $\bar{p}_2^*$. From 17, we can infer that for the region $(\bar{p}_1^*,\bar{p}_2^*)$ (Equation 10 in “Appendix A.1”), $\bar{p}_2^*(1-\bar{p}_2^*)w'(\bar{p}_2^*-)=\left[ rs+r\bar{p}_2^*\hat{g}+\bar{p}_2^*2\hat{g}-(r+\bar{p}_2^*)[\frac{\lambda }{\lambda _2}s - \frac{\hat{g}(1-\lambda _2)}{\lambda _2} \bar{p}_2^*]\right]$. Similarly, for the region $(\bar{p}_2^*,1)$, we find $\bar{p}_2^*(1-\bar{p}_2^*)w'(\bar{p}_2^*+)=\left[ (r+\lambda )\bar{p}_2^*2\hat{g}-(r+\lambda \bar{p}_2^*)[\frac{\lambda }{\lambda _2}s - \frac{\hat{g}(1-\lambda _2)}{\lambda _2} \bar{p}_2^*]\right] /\lambda$, and hence $w'(\bar{p}_2^*+)=w'(\bar{p}_2^*-)$.

It remains to show that *w* solves the Bellman equation, i.e. that $\bar{B}_i\le \bar{c}$ for both $i\in \{1,2\}$ on $[0,\bar{p}_1^*]$; $\bar{B}_1\ge \bar{c}$ and $\bar{B}_2\le \bar{c}$ on $(\bar{p}_1^*,\bar{p}_2^*]$; and $\bar{B}_i\ge \bar{c}$ for both $i\in \{1,2\}$ on $(\bar{p}_2^*,1]$. First, consider $p\in [0,\bar{p}_1^*]$. In this case, $w=2s$, and $\bar{B}_i\le \bar{c}$ if and only if $p\le \frac{rs}{r\hat{g}+\lambda _i(2\hat{g}-2s)}$, which is verified for all $p\le \bar{p}_1^*$. Now, consider $p\in (\bar{p}_1^*,\bar{p}_2^*]$. As *w* is strictly increasing in this range, $w=2s+\bar{B}_1-\bar{c}>2s$, and thus $\bar{B}_1>\bar{c}$. Moreover, $w=2s+\bar{B}_1-\bar{c}$ implies that $\bar{B}_2=\lambda _2\bar{B}_1=\lambda _2\left( w-s-p\hat{g}\right) \le s-p\hat{g}=\bar{c}$ if and only if $w\le \frac{\lambda }{\lambda _2}s - \frac{\hat{g}p[1-\lambda _2]}{\lambda _2}$, which is verified as $p\le \bar{p}_2^*$. Finally, let $p\in (\bar{p}_2^*,1)$. In this range, we have that $2\hat{g}-w-(1-p)w'=\frac{r}{\lambda p}\left( \frac{\lambda }{\lambda _2}s-\bar{p}_2^*2\hat{g}\right) \frac{u_0(p)}{u_0(\bar{p}_2^*)}$, so that $\bar{B}_2=\frac{\lambda _2}{\lambda }w-\frac{\lambda _2}{\lambda }2\hat{g} p$, which exceeds $\bar{c}=s-p\hat{g}$ if and only if $w\ge \frac{\lambda }{\lambda _2}s - \frac{\hat{g}p[1-\lambda _2]}{\lambda _2}$. By monotonicity of *w*, $w \ge \frac{\lambda }{\lambda _2}s - \frac{\hat{g}p[1-\lambda _2]}{\lambda _2}$ in this range, which implies $\bar{B}_2 \ge \bar{c}$. Also, $\bar{B}_1 = \frac{\bar{B}_2}{\lambda _2} > \bar{B}_2 \ge \bar{c}$. This completes the proof.

### B.9 Proof of Proposition 9

As for Proposition 3, we need to establish that $\bar{p}_2^{'} \ge \bar{p}_1^{'}$ if and only if $\lambda _2>\hat{\lambda }_2$, for some $\hat{\lambda }_2\in (0,1)$. As $\lambda _2$ increases, $\bar{D}_1$ ($\bar{D}_2$) continuously rotates clockwise (anticlockwise). As $\bar{w_i}$ ($i = 1,2$) are independent of $\lambda _2$, it follows immediately that $\bar{p}_1^{'}$ ($\bar{p}_2^{'}$) is increasing (decreasing) in $\lambda _2$. As $\lambda _2 \uparrow 1$, $\bar{D}_1$ and $\bar{D}_2$ coincide in the limit, and, since $\bar{w_2}>\bar{w_1}$ on $(\hat{p}_1,1)$, this implies $\bar{p}_2^{'} < \bar{p}_1^{'}$. On the other hand, as $\lambda _2 \downarrow 0$, $\bar{p}'_1\rightarrow \hat{p}_1$ and $\bar{p}'_2\rightarrow \frac{s}{\hat{g}}>\hat{p}_1$. This concludes the proof.

### B.10 Equations for subsection 4.5

Consider the case when $\lambda _1 > \lambda _2$, but $g_1 = g_2 = \hat{g}$.

*Planner’s problem*

The planner’s value function *w* satisfies

17 $$\begin{aligned} w(p) = 2s + \max _{k_1,k_2 \in \{0,1\}} \left\{ k_1 [\bar{B}_1(p,w)-\bar{c}(p) ] + k_2 [\bar{B}_2(p,w) - \bar{c}(p)]\right\} \end{aligned}$$

where

$$\begin{aligned} \bar{B}_i(p,w) = \frac{\lambda _i p[2\hat{g} -w(p) -w^{'}(p)(1-p)]}{r} \end{aligned}$$

and $$\bar{c}(p) = s - \hat{g} p$$.

*Non-cooperative game*

Player *i*’s value function $w_i$ satisfies

18 $$\begin{aligned} w_i(p) = s+k_j(p)\lambda _j \bar{b}_i(p,w_i) +\max _{k_i\in \{0,1\}} k_i [\lambda _i \bar{b}_i(p,w_i)- (s-\hat{g}p)] \end{aligned}$$

where

$$\begin{aligned} \bar{b}_i(p,w) = p\frac{\hat{g} - w_i(p)- (1-p)w_i^{'}(p)}{r} \end{aligned}$$

As before, we can derive the best response diagonals as

$$\begin{aligned} \bar{D}_i(p) = s+\frac{\lambda _j}{\lambda _i}[s-\hat{g}p] \end{aligned}$$

For beliefs right above $\hat{p_1}$, pay-offs to players 1 and 2 are given by

$$\begin{aligned} \bar{w_1}(p) = \hat{g} p + [s - \hat{g}\hat{p_1}]\frac{u_1(p)}{u_1(\hat{p_1})}, \end{aligned}$$

and

$$\begin{aligned} \bar{w_2}(p) = s + \frac{\hat{g}-s}{1+r}p - \frac{\hat{g}-s}{1+r}\hat{p_1}\frac{u_1(p)}{u_1(\hat{p_1})}, \end{aligned}$$

respectively.

## Footnotes

1 The first paper to do so was Bolton and Harris (1999). Keller et al. (2005) have introduced exponential bandits, which we shall use here.

2 See Hörner and Skrzypacz (2016) for an overview

3 Parameter values for this figure: $\lambda _1 = 1$; $\lambda _2 = 0.4$; $h = 3.5;s=1$ and $r = 0.9$. $p_1^{*} = 0.1488$ and $p_2^{*} = 0.6686$.

4 By standard results, on any open interval of beliefs in which player *j*’s action choice is constant, player *i*’s value function $v_i$ will be continuously differentiable. At those (finitely many) beliefs at which player *j*’s action changes, $v'$ should be understood as the left derivative of *v* (since beliefs can only drift down).

5 $\bar{v_1}$ and $\bar{v_2}$ are obtained from Eqs. 14 and 16, respectively, by imposing the condition $\bar{v_i}(\bar{p}_1) = s$ ($i=1,2$).

6 Parameter Values: $\lambda _1=1;\lambda _2 = 0.9$; $h = 2;s=1$ and $r = 1.2$. $p_1^{*} = 0.2857$ and $p_2^{*} = 0.4126$. $p_1^{'} = 0.4629$ and $p_2^{'} = 0.4916$.

7 These pay-offs are obtained from 12 by imposing the condition $v_i^r(p_2^{'}) = \bar{v_i}(p_2^{'})$ ($i = 1,2$).

8 All our figures correspond to parametric values such that the point of intersection of $v_2$ and $v_1$ lies to the right of $p_2^{'}$. However, for very low values of $\lambda _2$, this intersection will occur to the left of $p_2^{'}$. All of our analysis goes through unchanged for this case as well.

9 Parameter values: $\lambda _1 = 1;\lambda _2 = 0.985;h = 2;s = 1$ and $r = 1.9$. $\bar{p}_1 = 0.3958;p_1^{'} = 0.4754;p_2^{'} = 0.4644$ and $p_s^1 = 0.4740$.

10 For the particular parameter values used in Fig. 3, we have $v_1^{'}(p_2^{'-}) = 0.9694$; $v_1^{'}(p_2^{'+}) = 1.5085$; $v_2^{'}(p_2^{'-}) = 0.9894$; $v_2^{'}(p_2^{'+}) = 0.3197$.

11 For the particular parameter values used in Fig. 3, we have $v_2^{'}(p_s^{1-}) = 0.4625$; $v_2^{'}(p_s^{1+}) = 1.0721$.

12 The uniqueness of $p_S^n\in (\bar{p}_1,\frac{s}{g_n})$ follows from (11).

13
Dong (2018) shows that if the players’ initial beliefs are asymmetric enough, equilibrium welfare improves.

14 Proposition 6 implies that the qualitative characteristics of $p_s^1$ and $p_s^2$ are the same in any equilibrium in non-cut-off strategies. For simplicity, we consider an equilibrium in non-cut-off strategies where players swap roles only once in the figure.

15 Parameter values: $\lambda _1 = 1; \lambda _2 = 0.9;h;s=1$ and $r = 1.2$. $\bar{p}_1 = 0.3529;p_1^{'} = 0.4629;p_2^{'} = 0.4916;p_s^1 = 0.4499;p_s^2 = 0.5081$ and $\tilde{p_1} = 0.39$.

16 Mathematically, this can be seen as follows: consider a function $v = g_2 p + C(1-p) (\frac{1-p}{p})^{\frac{r}{\lambda _2}}$, such that the integration constant is derived from $v(\tilde{p_1}) = s$. Since $\tilde{p_1}<\bar{p}_2$, direct computation shows that $v^{'}(\tilde{p_1}) < 0$. In the equilibrium in non-cut-off strategies, to the immediate right of $\tilde{p_1}$, 2’s pay-off is given by $v_2 = g_2p +c_2(1-p) (\frac{1-p}{p})^{\frac{r}{\lambda _2}}$. The integration constant $c_2$ is determined from $v_2(\tilde{p_1}) = \bar{v_2}(\tilde{p_1})>s \Rightarrow c_2 > C$. Direct computation shows that this implies that 2’s pay-off will be negatively sloped in some right neighbourhood of $\tilde{p_1}$.

17 Please refer to “Appendix (B.10)” for explicit expressions for these functions.

18 Parameter values: $\lambda _1 = 1; \lambda _2 = 0.3;h_1 = 2;h_2 = \frac{20}{3};s=1$ and $r = 1.2$. $\hat{p_1} = 0.3529;\bar{p}_1^{'} = 0.4344;\bar{p}_2^{'} = 0.4779$.

19 Parameter values: $\lambda _1=\lambda _2 = 1$; $h_1=2.1;h_2= 1.9$; $s=1$ and $r=1.2$. $\tilde{p_1} = 0.3315;\tilde{p_1^{'}} = 0.4418$ and $\tilde{p_2^{'}} = 0.4582$.
